# Differential Effects on Survival, Humoral Immune Responses and Brain Lesions in Inbred BALB/C, CBA/CA, and C57BL/6 Mice Experimentally Infected with *Neospora caninum* Tachyzoites

**DOI:** 10.5402/2013/830980

**Published:** 2013-03-24

**Authors:** Tanja Mols-Vorstermans, Andrew Hemphill, Thierry Monney, Dick Schaap, Eveline Boerhout

**Affiliations:** ^1^Microbiology R&D, MSD Animal Health, Boxmeer, The Netherlands; ^2^Institute of Parasitology, Vetsuisse Faculty, University of Bern, 3012 Bern, Switzerland

## Abstract

C57BL/6, BALB/c, and CBA/Ca mouse strains with different MHC-I haplotypes were compared with respect to susceptibility to *Neospora caninum* infection. Groups of 5 mice received 1 × 10^6^, 5 × 10^6^, or 25 × 10^6^ tachyzoites of the NC-Liverpool isolate by intraperitoneal injection and were observed for disease symptoms. Humoral responses, splenocyte interferon-*γ* (IFN-*γ*) production, cerebral parasite loads, and histopathology were evaluated at human end points or the latest at 34 days postinfection (PI). The mortality rates in C57BL/6 mice were the highest, and relatively high levels of IgG1 antibodies were detected in those mice surviving till 34 days PI. In lymphocyte proliferation assays, spleen cells from C57BL6 mice stimulated with *N. caninum* antigen extract exhibited large variations in IFN-*γ* production. In BALB/c mice mortality was 0% at the lowest and 100% at the highest infection dose. Serologically they responded with high levels of both IgG2a and IgG1 subclasses, and lymphocyte proliferation assays of surviving mice yielded lower IFN-*γ* levels. CBA/Ca mice were the most resistant, with no animal succumbing to infection at a dose of 1 × 10^6^ and 5 × 10^6^ tachyzoites, but 100% mortality at 25 × 10^6^ tachyzoites. High IgG2a levels as well as increased IFN-*γ* in lymphocyte proliferation assays were measured in CBA/Ca mice infected with 1 × 10^6^ tachyzoites.

## 1. Introduction

*Neospora caninum* is an apicomplexan protozoan, infecting a large range of mammals. In cattle, this parasite represents a major cause of abortion [1–3]. Several studies focussing on the specific immune response to *N. caninum *in cattle have shown that the time of gestation is important with regard to the outcome of the infection. This may be explained on the basis of hormone levels and cytokine profiles [4]. Proinflammatory cytokines, produced by lymphocytes, are crucial for controlling a variety of intracellular pathogens, including *N. caninum*. These cytokines are produced by natural killer (NK) cells, as well as by CD4^+^  T-cells and CD8^+^  T-cells. CD4^+^ cells mediate the humoral response and their involvement is associated with increased IgG1 levels, whereas CD8^+^ cells are involved in the cellular immune response, which is characterized by increased production of IgG2a. Indeed, both has been observed in experimentally and naturally infected animals [5]. 

*N. caninum *is an intracellular parasite and resides within a specialized compartment, a parasitophorous vacuole, surrounded by a parasitophorous vacuole membrane (PVM). Following egress from a host cell, these parasites immediately search for a new host cell to invade, and the direct accessibility for components of the immune system to the parasite within the circulation is rather limited. Thus, extracellular tachyzoites have a low chance to be detected by T-helper-2-(Th2-) type cells. A humoral immune response is therefore not sufficient to clear a *N. caninum *infection. For more efficient protection against intracellular pathogens such as *N. caninum*, the host usually generates a cellular, T-helper-1-(Th1-) type response [6, 7]. This is possible since secretory parasite molecules pass through the PVM into the host cell cytoplasm, where they interact with and manipulate host cell functions [8]. Once in the cytoplasm these molecules can be processed and corresponding peptides are presented on the host cell surface via major histocompatibility complex (MHC) class I molecules. CD8^+^ lymphocytes will recognize the peptides presented on these MHC-I molecules and activate Th1 cells, which then produce cytokines such as interleukin-12 (IL-12), interferon gamma (IFN-*γ*), and tumour necrosis factor *α* (TNF-*α*). The production of these cytokines leads to the activation of pathways that generate free oxygen radicals and nitric oxide (NO) and its metabolites among other factors, which are potentially lethal for many protozoa [9].

However, these processes can also be deleterious to the fetoplacental interface and potentially induce abortion and/or fetal resorption [10], especially in the first trimester of pregnancy, when levels of pregnancy hormones, such as progesterone, which counteracts these effects, are relatively low. As a result, there is no or only little Th2 cytokine polarization and the dam will generate a Th1 immune response, which also affects the placental and foetal tissue. Since a strong Th1 response is incompatible with successful pregnancy, the infection can lead to the loss of the unborn foetus. If the infection occurs in the third trimester, progesterone levels are high. Progesterone promotes Th2 cell proliferation the production of IL-4, IL-5, and IL-10 is known to inhibit NO and TNF-*α* production and impairs NK cell activity [11–13]. The risk of abortion due to a Th2 response is therefore relatively low. However, since the immune response is not capable of controlling the parasite, the risk of transplacental transmission of *N. caninum* is relatively high [14]. Thus, resistance to *N. caninum* seems to rely, at least partially, on a functional Th1 response. 

In this study, inbred BALB/c, CBA/Ca, and C57BL/6 strains of mice (with different MHC-I haplotypes) were compared for their capacity to cope with a *N. caninum* infection. We found different outcomes of infections in the three models. In addition, we show that CBA/Ca mice were the by far most resistant, and protection correlated with the induction of a Th1-biased response. The results demonstrate that a meaningful evaluation of the efficacy of, for example, vaccine candidates and/or chemotherapeutically interesting compounds against experimental infection with *N. caninum* in mice requires standardization with regard to the mouse strains used for such experiments. 

## 2. Materials and Methods

### 2.1. Mice and Housing Conditions

Female BALB/c, CBA/Ca, and C57BL/6 mice aged 8–12 weeks were obtained from Harlan (Horst, The Netherlands). Mice were randomly divided into groups as soon as they came to hand and marked individually. The conditions in the animal housing facilities were negative air pressure, air exchange of 300 m^3^/hr, temperature of 22°C ± 2°C, and artificial day/night cycle with 12 hrs light per day. Five mice were housed per group in Macrolon Type III cages (820 cm^2^) enriched with polyvinyl chloride tubes and tissues. Tissues and Type GS bedding material were refreshed at least once per two weeks. Mice received *ad libitum* standard rodent feed (RMHB2118, AB-diets Woerden, The Netherlands). Water was supplied in bottles and refreshed at least three times a week. Prior to challenge, mice were given an acclimatization period of 7 days, during which they were observed daily for normal behaviour, wellness, food and water intake, and posture. 

### 2.2. *N. caninum* Tachyzoite Culture

*N. caninum* tachyzoites (NC-Liv isolate) were maintained by continuous passages in Vero cells grown in RPMI 1640 medium supplemented with 10% foetal bovine serum (FBS), 2 mM L-glutamine and 25 mg/L gentamycin sulphate. Cultures were grown in a humidified incubator at 37°C with 5% CO_2_. To liberate the tachyzoites from the Vero cells, the culture was taken up through a 20G needle and extruded through a 26G needle. For the preparation of parasites for infection, tachyzoites were purified on a PD10 column (GE Healthcare, Diegem, Belgium) centrifuged and resuspended in sterile 0.04 M isotonic PBS. Tachyzoites were counted in a Neubauer chamber. In order to prepare *N. caninum* tachyzoite antigen lysate, purified tachyzoites were subjected to one freeze-thaw cycle followed by sonication on a Branson Sonifier S-250A (Branson ultrasonics, Danbury, USA) for 60 sec, output 4, and a cycle duty of 50%. The antigenic solution was divided into aliquots and stored at −20°C until further use.

### 2.3. Experimental Infection of Mice and Subsequent Monitoring of the Infection

Mice were challenged by intraperitoneal injection of 1 × 10^6^, 5 × 10^6^, or 25 × 10^6^ live NC-Liv tachyzoites in 0.5 mL 0.04 M isotonic PBS. Mice were then closely monitored daily for neosporosis disease symptoms according to a clinical scoring system that included hunched back, rough hair coat, impaired movement, and spinning. For each feature a mouse obtained a score of 1, the maximal score being 4. The average clinical score per group was determined by dividing the total score per group by the amount of mice in that group. As soon as the health status of the animals deteriorated, the frequency of observations was increased to two times a day. Weight loss was measured three times a week during the entire experimental period. Moribund mice or mice with severe neurobiological symptoms were euthanized (humane endpoint).

Blood samples were collected by orbital puncture at day 0 and day 34 postinfection (at the time of euthanasia) or, whenever possible, at the time of inter-current death. Prior to blood sampling and euthanasia, mice were subjected to anaesthesia by isoflurane. Latest at day 34 postinfection (PI), surviving mice were sacrificed by cervical dislocation and brains and spleens were harvested. For each mouse, one brain hemisphere was stored at −20°C for further DNA extraction. The other half was fixed in 10% neutral buffered formalin for histopathology. Spleens were immediately processed as described below.

### 2.4. Histopathology

Formalin-fixed material was processed into paraffin wax. Sections of 3.5 *μ*m thickness were cut longitudinally from nervus I to medulla oblongata with cerebrum, hippocampus, and cerebellum on a Brand Microm H355 S microtome (Adamas, Rhenen, The Netherlands), and were placed onto glass slides. Sections were stained with haematoxylin-eosin.

### 2.5. Serological Analysis

Serum antibody levels were analysed using a commercially available ELISA kit based on antigens of *N. caninum* (IDEXX *Neospora *Ab Test, IDEXX Laboratories, Westbrook, USA) according to the instructions of the manufacturer. On each plate, sera were tested in 2log titrations, and negative and positive sera were tested in octaploid. Sera of naïve Swiss outbred mice (Charles River, Sulzfeld, Germany) were used as a negative control. As a positive control for IgG1 and IgG2a detection, sera of BALB/c mice vaccinated with the chimeric antigen R-MIC3-1 and challenged with *N. caninum *(NC-1 isolate) tachyzoites were used [2, 3]. Sera of C57BL/6 mice taken 34 days after infection with 5 × 10^6^  NC-Liv tachyzoites were used as a positive control for IgG2c analysis. Horseradish-peroxidase-(HPR-) conjugated goat antimouse IgG1, IgG2a, and IgG2c were obtained from Southern Biotechnology (Birmingham, USA) and diluted in enzyme immune assay (EIA) buffer containing 0.05% polysorbate 80. Results were analysed on a Tecan SUNRISE device (Breda, The Netherlands) using XFluor4 Software at 650 nm. Antibody titres were determined using CaSpEx Software; AbendVertical version 0.11 V1 (MSD Animal Health, Proprietary Software). The cutoff in antibody end titres was defined at B_min_∗2, where B_min_ is the negative control.

### 2.6. Lymphocyte Proliferation Assay and Cytokine Measurements

Spleens from surviving mice were washed twice in RPMI 1640 medium supplemented with 100.000 IU penicillin/streptavidin, 1 mM sodium-pyruvate, and 2 mM L-glutamine and dissociated using the gentleMACS Dissociator (Miltenyi Biotec, Leiden, The Netherlands) according to the instructions of the manufacturer. Suspensions were filtered through a 100 *μ*m cell strainer and centrifuged at 300 ×g for 10 min at 4°C. Samples were depleted of erythrocytes with erythrocyte lysis solution containing 829 g/L ammonium chloride, 100 g/L sodium hydrogen carbonate, and 3.7 g/L disodium edetate. Splenocytes from mice of the same group were pooled in a 1 : 1 ratio and a total of 1 × 10^5^ splenocytes were cultured for 72 hrs in 100 *μ*L complete RPMI medium containing 10% FBS in a humidified incubator at 37°C with 5% CO_2_. 100 *μ*L of an antigenic extract of either 2 × 10^4^, 1 × 10^5^, or 5 × 10^5^ NC-Liv tachyzoites prepared as described above was added to the cells. Complete RPMI medium and a phorbolmyristateacetate (PMA)/ionomycin mixture (9.74 ng/mL/0.26 mM) were used as negative and positive controls, respectively. Cytokine levels in supernatants of stimulated splenocytes were analyzed using the BenderMedSystems FlowCytomix Mouse/Rat Basic Kit combined with the cytokine Mouse Simplex Kits for IFN-*γ* (BenderMedSystems,Vienna, Austria). Cytomix assays were performed according to the instructions of the manufacturer. Results were analyzed using the BenderMedSystems FlowCytomix Pro 2.4 Software. The samples of the cytomix assay were interpolated in the standard curve by selecting the *5P logistic fit* function: the BenderMedSystems FlowCytomix Pro 2.4 Software fits the best curve according to *y* = *d* + ((*a* − *d*)/(1 + (*x*/*c*)^*b*^)^*g*^). The mean fluorescence intensity (MFI) of each standard point is blank-corrected by division of the Blank-MFI (B_B0 = MFI/MFI of Blank∗100). The maximum acceptable bias, which displays the variation for the ideal standard curve defined by the theoretical standard concentrations, was set at 30%. 

### 2.7. PCR Analysis in Brain Tissue

*Neospora*-specific quantitative real-time PCR to determine the number of tachyzoites that has reached the cerebral tissue was performed as previously described [2, 3, 15]. DNA extraction from brain tissue was performed using the DNeasy Blood & Tissue Kit (Qiagen, Hilden, Germany) according to the manufacturer recommendations. The DNA concentration in each sample was determined by UV spectrophotometry (NanoDrop, Thermo Scientific, Delaware, USA) and was adjusted to 5 ng/*μ*L with sterile DNase-free water. Quantitative real-time PCR was performed using the Light Cycler Instrument (Roche Diagnostic, Basel, Switzerland). The parasite counts were calculated by interpolation from a standard curve with DNA equivalents from 1000, 100, and 10 parasites included in each run.

### 2.8. Statistics

Mice survival was analyzed according to Kaplan-Meier and the survival curves between groups were compared with the logrank test followed by regression coefficient analysis. The weight of the survivors, cerebral parasite burden, lesion score, and the serological data were compared using Kruskal-Wallis one-way ANOVA followed by the Kruskal-Wallis multiple comparison *Z*-value test. The *P* value between two significantly different groups was calculated by Mann-Whitney *U* test. The proportion of animals with clinical signs, proportion of animals with brain lesions and data on *N. caninum*-DNA-positive animals were organized in a contingency table and compared by a Chi-square test.

## 3. Results

### 3.1. Clinical Observations

**Figure 1:**
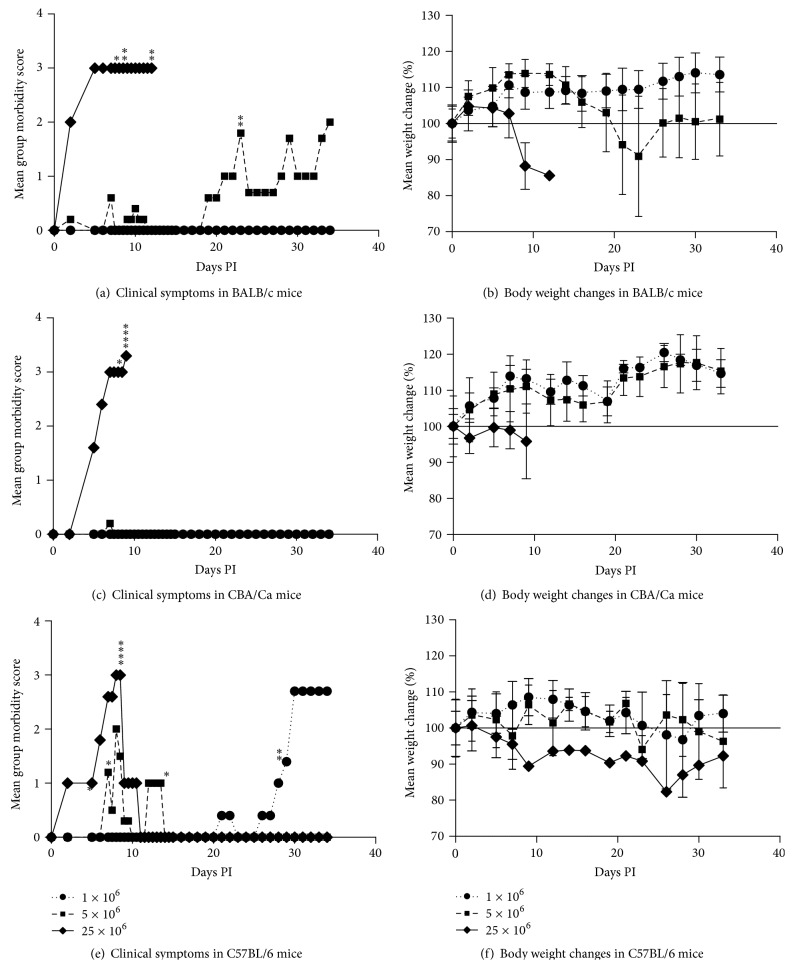
Mean group morbidity scores ((a), (c), and (e)) and mean weight changes ((b), (d), and (f)) in BALB/c, CBA/Ca, and C57BL/6 mice following i.p. inoculation with 1 × 10^6^, 5 × 10^6^, or 25 × 10^6^  NC-Liv tachyzoites. Mice were scored for clinical symptoms including hunched back, ruffled hair coat, impaired movement, and spinning when picked up by the tail, resulting in a maximum clinical score per mouse of 4. Figures 1(a), 1(c), and 1(e) show the average clinical score per mice per group. ∗indicate the death of a mouse; error bars ± SD; *n* = 5.

BALB/c mice (Figures 1(a) and 1(b)) challenged with 1 × 10^6^ tachyzoites were not affected, with no decrease in body weight following infection. However, challenge with 5 × 10^6^  NC-Liv tachyzoites resulted in neosporosis symptoms in BALB/c mice, starting on day 18 PI. Hunched back, ruffled coat, and weight loss of ~25% were observed in these mice. Two mice reached the point where severe neurobiological symptoms made it necessary to get them euthanized on day 23 PI, while the other three mice, although exhibiting clinical symptoms, but less severe, survived throughout the observation period of 34 days (Figures 1(a) and 1(b)). BALB/c mice were not able to control the highest infectious dose of 25 × 10^6^ tachyzoites, resulting in death of all five animals within that group between days 7 and 12 PI. 

**Figure 2:**
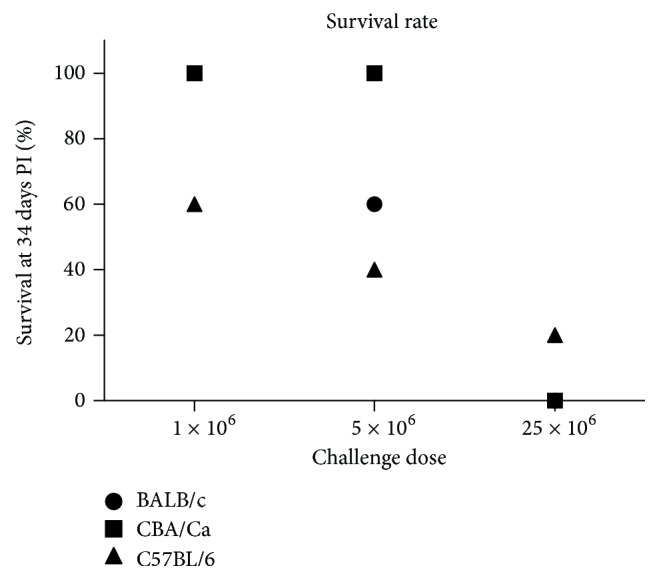
Survival plot of mice 34 days after challenge with NC-Liv tachyzoites.

CBA/Ca mice (Figures 1(c) and 1(d)) exhibited a high degree of resistance against *N. caninum* at an infection dose of 1 × 10^6^ and 5 × 10^6^ tachyzoites, with no mortality and no body weight changes during the entire experimental period (Figures 2 and 1(d)). However, none of the CBA/Ca mice could control the infection at the highest dose and they all were euthanized within 9 days PI (Figures 1(c) and 1(d)).

In comparison to the other two mouse strains, C57BL/6 mice (Figures 1(e) and 1(f)) were the most sensitive. At an infection dose of 1 × 10^6^ tachyzoites, all mice started to exhibit clinical symptoms including weight loss of ~10% and two out of five mice had to be euthanized at day 29 PI (Figure 2). Strong variations were observed in the group of C57BL/6 mice challenged with 5 × 10^6^ tachyzoites. Three mice of this group demonstrated severe symptoms of neosporosis and were euthanized on days 7, 8 and 13 PI, while the other two mice showed only very mild symptoms on day 8 PI and returned to being clinically normal 9 days after the challenge. Shortly after a challenge with 25 × 10^6^ tachyzoites, C57BL/6 mice showed a hunched back and ruffled hair coat (Figures 1(e) and 1(f)). Symptoms progressed to include impaired movements and spinning when picked up by the tail, requiring euthanasia of four of these mice on day 8 PI. The remaining mouse of this group completely recovered by day 11 PI and survived till the end of the experiment. 

Statistically, CBA/Ca mice exhibited a significantly higher survival rate than C57BL/6 mice at an infection dose of 1 × 10^6^ tachyzoites (*P* < 0.001, Cox regression analysis) and than C57BL/6 and BALB/c mice at an infection dose of 5 × 10^6^ tachyzoites (*P* < 0.001, Cox regression analysis). The mean weight of the survivors of the BALB/c mice infected with 1 × 10^6^ tachyzoites was slightly higher than those infected with 5 × 10^6^ parasites, although the difference was not significant (Kruskall-Wallis multiple comparison test). The number of symptomatic mice in the CBA/Ca mice at an infection dose of 5 × 10^6^ tachyzoites was significantly lower than in the two other groups (*P* < 0.01, Chi-square test) while no significant differences between strains were observed for the other infection doses (Table 2). The number of mice presenting brain lesions as well as the lesion score per group at an infection dose of 5 × 10^6^ parasites was significantly lower in the CBA/Ca mice (*P* < 0.01, Chi-square test and *P* < 0.001, Mann-Whitney *U*-test, resp.) than in the two other groups (Table 2). No significant difference was observed regarding the overall lesion score per strain (Table 2). Moreover, C57BL/6 mice showed strong variations within each group. 

The surviving BALB/c mice infected with 1 × 10^6^ tachyzoites (*n* = 5) and CBA/Ca mice infected with 5 × 10^6^ tachyzoites (*n* = 5) were challenged again with 25 × 10^6^ *N. caninum* tachyzoites at day 34. However, they all succumbed to infection within 24 hrs, indicating that they had not built up any form of protective immunity. 

### 3.2. Serology

In order to obtain information on the type of immune response induced by *N. caninum* infection, the serum titres of *N. caninum*-specific IgG1 and IgG2a antibodies were recorded. The production of IgG1-type antibodies is primarily induced by a humoral immune response, whereas IgG2a subclasses indicate the involvement of a cellular immune response. Since C57BL/6 mice poorly produce IgG2a antibodies, the IgG2c levels were measured in serum of these mice. In none of the groups *N. caninum-*specific antibodies were detected prior to challenge (data not shown). Experimental infection of BALB/c mice with 1 × 10^6^ or 5 × 10^6^ tachyzoites resulted in high IgG2a and IgG1 serum levels, indicating the presence of a mixed cellular and humoral immune response (Table 1). In CBA/Ca mice challenged with 1 × 10^6^ or 5 × 10^6^ tachyzoites, predominantly IgG2a antibodies were detected. IgG2a and IgG1 were undetectable in CBA/Ca mice challenged with 25 × 10^6^  tachyzoites, most likely because mice succumbed to the infection before a humoral response was mounted (Table 1). C57BL/6 mice exhibited high variations in IgG1 and IgG2c serum levels. In general, sera of mice surviving until day 34 PI contained higher levels of IgG2c compared to sera of their group mates that died at earlier time points (Table 1). The difference IgG2a/c-IgG1 postinfection was highly significantly higher in the CBA/Ca mice than in the two other strains (*P* < 0.01, Mann-Whitney *U*-test) and significantly higher in the C57/BL6 mice than in the BALB/c mice (*P* < 0.05, Mann-Whitney *U*-test) at an infection dose of 1 × 10^6^ parasites. At an infection dose of 5 × 10^6^ tachyzoites, both CBA/Ca and C57/BL6 mice had a significantly higher IgG2a/c-IgG1 difference than the BALB/c mice (*P* < 0.01, and *P* < 0.05 respectively, Mann-Whitney *U*-test), and CBA/Ca mice also had a higher IgG2a/c-IgG1 difference than C57/BL6, although not significant (Table 2 and Figure 4).

### 3.3. Stimulation of Spleen Cells of Selected Survivors and IFN-*γ* Production upon Recall Responses

**Figure 3:**
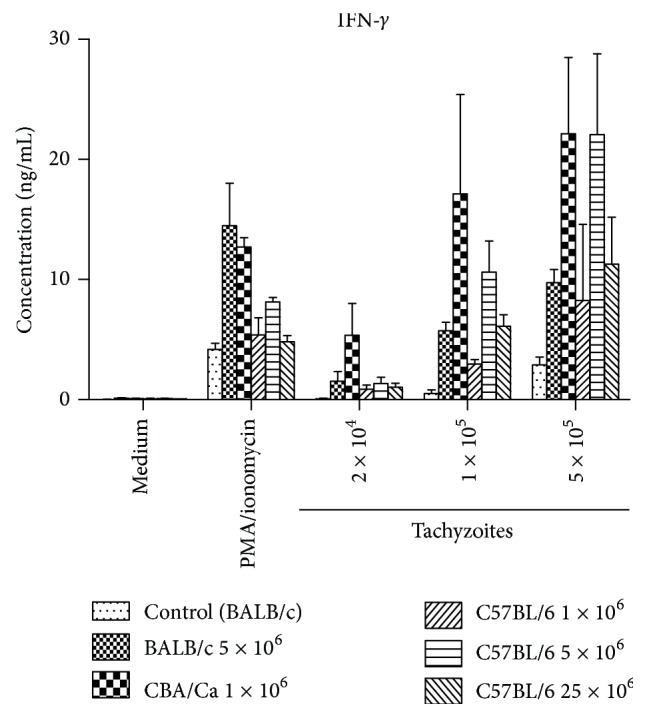
IFN-*γ* production by stimulated splenocytes derived from BALB/c, CBA/Ca, and C57BL/6 mice challenged with varying doses of Nc-Liv tachyzoites. The IFN-*γ* production was analyzed in triplicates. Results show the mean values ± SD.

**Figure 4:**
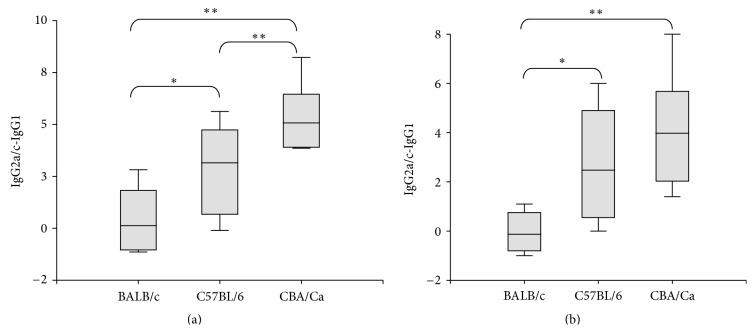
Difference between IgG2a or IgG2c and IgG1 levels in postinfection sera. (a) Infection dose of 1 × 10^6^ tachyzoites. (b) Infection dose of 5 × 10^6^ tachyzoites. ∗Indicates a statistically significant difference between the groups (*P* < 0.05). ∗∗Indicates a highly significant difference between the groups (*P* < 0.01).

The production of IFN-*γ*, indicating the occurrence of a cellular immune response, was measured in the supernatants of spleen cells derived from survivors of BALB/c mice (infected with 5 × 10^6^ tachyzoites), CBA/Ca mice (infected with 1 × 10^6^ tachyzoites), and all surviving C57BL/6 mice. Splenocytes were stimulated with either *N. caninum* antigens or PMA/ionomycin for 72 hrs, or were left unstimulated. Stimulation with a lysate corresponding to 2 × 10^4^, 1 × 10^5^ or 5 × 10^5^ purified NC-Liv tachyzoites resulted in increased production of IFN-*γ*. IFN-*γ* production was a dose-dependent stimulus in all groups (Figure 3). The strongest IFN-*γ* response was observed for the CBA/Ca mice, as was especially evident using low and medium tachyzoite stimulus doses. A lower IFN-*γ* production was observed for the BALB/c mice, while splenocytes from C57BL/6 mice demonstrated large variations in IFN-*γ* production upon antigen stimulation. Data from BALB/c mice and CBA/Ca mice infected with 25 × 10^6^ parasites is missing, since these mice succumbed to infection prior to 34 days postinfection. Spleen cells of BALB/c mice infected with 1 × 10^6^ tachyzoites (*n* = 5) and CBA/Ca mice infected with 5 × 10^6^ tachyzoites (*n* = 5), which were used for rechallenge, were not assessed.

### 3.4. PCR Analysis in Brain Tissue and Comparison with Histopathology

The cerebral parasite load in all groups was measured by real-time PCR and compared with histopathological findings. Corresponding results are summarized in Table 2. In three out of five BALB/c mice challenged with 1 × 10^6^ tachyzoites, no parasite DNA could be detected by PCR, while high numbers were detected in the other two samples. However, in only one of these mice a brain lesion was detected by histology. Challenge of BALB/c mice with 5 × 10^6^ tachyzoites resulted in high cerebral parasite load in all mice, and brain lesions were detected upon histopathological inspection in four out of five mice. After infection of BALB/c mice with 25 × 10^6^ tachyzoites, parasites were detected at moderate to high numbers in all brain samples investigated, but histopathology failed to detect any lesions at all. 

Real-time PCR detected *N. caninum* DNA in one of 10 brain samples of CBA/Ca mice challenged with 1 × 10^6^ and 5 × 10^6^ tachyzoites. In contrast, lesions were observed by histopathological means in two mice infected with 1 × 10^6^ tachyzoites. However, these were samples that were not positive by real-time PCR. In the CBA/Ca mice infected with 25 × 10^6^ tachyzoites, lesions in the central nervous system (CNS) were observed by histopathology for three out of five animals, and real-time PCR showed high numbers of parasite load in all 5 members of the group, which all died between days 8 and 9 PI. 

In three C57BL/6 mice challenged with 1 × 10^6^ tachyzoite lesions in the brain were observed. In two of them parasites were measured 29 days PI. Infection with 5 × 10^6^ tachyzoites resulted in parasites and lesions in three mice on days 13 and 34 PI. In the C57BL/6 mice infected with 25 × 10^6^ tachyzoites, four out of five mice died 8 days after challenge, with real-time PCR-positive brain tissue but no lesions detectable upon histopathology. One mouse survived until day 34 PI, and this mouse exhibited a high cerebral parasite load and also brain lesions. Thus, a high cerebral parasite load as determined by real-time PCR did not always correspond with the detection of lesions in the brain and did also not always match with the occurrence of clinical signs of neosporosis. At an infection dose of 1 × 10^6^ and 25 × 10^6^ tachyzoites, no significant difference in the number of PCR positive mice between the groups was observed (Chi-square test). At an infection dose of 5 × 10^6^ tachyzoites, the number of PCR-positive mice was significantly lower in the CBA/Ca mice than in the BALB/c and C57/BL6 mice (*P* < 0.01 and *P* < 0.05, respectively, Chi-square test). No significant difference regarding the parasite load of infected mice was observed between the groups (Kruskal-Wallis multiple comparison test).

## 4. Discussion

In this study, we compared the capacities of three inbred mouse strains expressing different MHC-I molecules to cope with a *N. caninum *infection. Significant differences in the responses to *N. caninum* infection in inbred and outbred mice have been demonstrated previously [16], and these differences have been postulated to be due to varying abilities to present *N. caninum *antigens on their MHC molecules [17]. When using mouse models to investigate the outcome and immunological parameters of infection, the selection of the *N. caninum* isolate as well as inoculum doses are important variables that will influence the results [16]. The present study showed high variations in morbidity and mortality in the three inbred mouse strains BALB/c, CBA/Ca, and C57BL/6 after inoculation with different numbers of *N. caninum* (NC-Liv) tachyzoites, demonstrating that the choice of mouse strain plays a crucial role for the assessment of experimental infections and agents that could potentially interfere therein. 

While BALB/c mice did not suffer from clinical signs of neosporosis after a challenge dose of 1 × 10^6^ *N. caninum* tachyzoites, infection with 5 × 10^6^ tachyzoites resulted in severe clinical symptoms starting at day 20 PI. Also C57BL/6 mice were highly susceptible to experimental infection with *N. caninum* tachyzoites. This is in accordance with several vaccination studies, which have used the BALB/c and C57BL/6 models to investigate the protective effects of vaccine candidates (reviewed in [2, 3]). In contrast, only limited research has been performed using CBA/Ca mice as a model to investigate the immune responses against *N. caninum *infection. Our investigations demonstrated that this mouse strain is clearly more resistant against neosporosis compared to BALB/c and C57BL/6 mice. Previously, these features of CBA/Ca mice with regard to *N. caninum* infection had been highlighted by Rettigner et al. [18], who described this mouse strain to be resistant against an infection of 5 × 10^6^ tachyzoites of the NC-1 isolate of *N. caninum* following treatment with immunosuppressing agents and suitable for the generation of cerebral tissue cysts. This is surprising, since the NC-1 isolate, in contrast to NC-Liv, does hardly convert into bradyzoites under *in vitro* conditions such as high pH treatment or incubation with sodium nitroprusside [19, 20].

With respect to C57BL/6 mice, Ramamoorthy et al. [21] observed no clinical signs of neosporosis throughout an observation period of 21 days after an infection with 5 × 10^6^  NC-1 tachyzoites but also showed these mice to be highly susceptible to an infection with 2 × 10^7^ NC-1 tachyzoites. Others failed to observe clinical signs in C57BL/6 mice after a challenge with 5 × 10^6^  NC-1 tachyzoites as long as 44 days PI [22]. In this study, after a challenge dose of 1 × 10^6^  NC-Liv tachyzoites disease symptoms occurred after 26 days but only after 8 days when 5 × 10^6^ tachyzoites were inoculated, with three out of five mice succumbing to infection. This illustrates that, besides the mouse strain used, the selection of the *Neospora *isolate also makes a big difference. In any case, an infection dose of 25 × 10^6^ tachyzoites of the NC-Liv isolate was far too high for any of the mouse strains to cope with, requiring euthanasia of all but one mouse between days 7–12 PI. 

Since *N. caninum *is an intracellular parasite, the protective immune response is likely to involve cell-mediated immunity; thus Th1-mediated responses are important. However, in BALB/c mice it has been demonstrated that besides cellular immunity also humoral immune responses are crucial in controlling *N. caninum* infection and limiting the pathological changes caused by an excessive proinflammatory response [23, 24]. Teixeira et al. [25] described that intraperitoneal injection of *N. caninum* tachyzoites (NC-1 isolate) in BALB/c mice induced a parasite-specific, nonpolyclonal, B-cell response. A strong bias towards a Th2-type response resulted in increased susceptibility to *N. caninum* and enhanced the corresponding pathologic effects [26]. However, vaccination of mice with recombinant rhoptry antigen ROP2 emulsified in saponin adjuvants induced an IgG1-biased humoral immune response, while formulating the same antigen in Freund's incomplete adjuvants resulted in an IgG2a-biased antibody response, and both were protective in the nonpregnant BALB/c mouse model. Protection associated with an IgG2a-biased humoral immune response was also demonstrated for recombinant protein disulfide isomerase applied for vaccination intranasally [27–29]. Vaccination of mice with native NcSRS2 induced a Th2-biased protective response that reduced congenital infection of offspring BALB/c mice [30]. Reduction in congenital infection, also associated with a Th2-biased immune response, was demonstrated by vaccination experiments in BALB/c mice employing combined NcMIC1, NcMIC3, and NcROP2 antigens [31]. Taken together, it is probably rather a suitable balance in Th1/Th2-type immune responses than a strict Th1 response that allows the host to deal with *N. caninum* infection [23]. 

Similar to earlier studies in BALB/c mice [25, 26], levels of IgG1 antibodies in animals infected with 1 × 10^6^ and 5 × 10^6^ tachyzoites were generally high, and it is interesting to note that a challenge dose of 5 × 10^6^ tachyzoites in BALB/c mice resulted in more pronounced IgG1 responses compared to the IgG1 antibody levels found in mice inoculated with 1 × 10^6^ tachyzoites, while IgG2a titres were basically equal in both groups. In the BALB/c mice inoculated with 5 × 10^6^ tachyzoites, titres for IgG1 and IgG2a were similar, which is in agreement with previous observations reported by Lundén et al. [32]. In addition, we found that splenocytes obtained from BALB/c mice inoculated with 5 × 10^6^ tachyzoites and stimulated with *N. caninum* antigen produced IFN-*γ*, albeit at a lower level compared to CBA/Ca mice. The results of Rettigner et al. [18] on *N. caninum *infection in CBA/Ca mice showed no evidence of differential isotype secretion but a high expression of IFN-*γ*, while in our experiments sera of infected CBA/Ca mice exhibited a high IgG2a response, and splenocytes of CBA/Ca mice infected with 1 × 10^6^ *N. caninum* tachyzoites also secreted high levels of IFN-*γ*. This indicated that the induction of a cellular immune response is a prerequisite for successful dealing with the infection.

The importance of the humoral immune response during *N. caninum* infection was demonstrated by Eperon et al. [22] who showed increased susceptibility to *N. caninum* (NC-1 isolate) infection in *μ*MT B-cell-deficient mice when compared to wild-type C57BL/6 mice. They also described the predominant presence of parasite-specific IgG2a isotypes and an absence of IgG1 isotypes in C57BL/6 mice at various time points. In contrast to Eperon et al., we did not observe any IgG2a antibody titres in C57BL/6 mice and analysed the IgG2c antibody isotype levels instead. Our results showed the major parasite-specific IgG isotype to be IgG2c. In agreement with a shift from a Th1- to a Th2-biased response, increasing titres of *N. caninum*-specific IgG1 were detected in mice sera collected at later time points after infection. However, in our hands the C57BL/6 mice exhibited considerable variability.

While *N. caninum *obviously induces differential humoral and cellular immune responses in different mouse strains during the acute phase of infection, one could imagine that chronic infection would be characterized by the production of tissue cysts. Vertical transmission in cattle is mediated by recrudescence of tissue cysts. Therefore, in order to mimic the infection in cattle, a mouse model able to form tissue cysts, and also exhibiting recrudescence during pregnancy, is required. However, only few reports have actually been published on the production of *N. caninum* tissue cysts in mice. Tissue cyst production had been demonstrated in outbred mice and immunosuppressed CBA/Ca mice inoculated with NC-1 or NC-Liv tachyzoites but not all of the mice investigated harboured cerebral tissue cysts and the rate of mortality was high (31%) [33]. Rettigner et al. [18] showed that immunosuppressed female CBA/Ca mice given 5 × 10^6^  NC-1 tachyzoites were able to survive and to consistently develop cerebral tissue cysts. More recently, tissue cyst production, marked by positive staining with an antibody directed against the *Toxoplasma *bradyzoite antigen BAG5, has also been demonstrated in an experimentally infected carnivorous marsupial, the fat-tailed dunnart *Sminthopsis crassicaudata *[34]. It is also very likely that *N. caninum* tissue cysts are not necessarily formed predominantly in the CNS but also at other locations such as muscle tissue [34, 35], and this should be taken into account in future studies. 

In conclusion, we comparatively assessed BALB/c, CBA/Ca, and C57BL/6 mice as models for *N. caninum *infection and demonstrated that CBA/Ca mice exhibited the highest degree of resistance at low and medium infection doses but rapidly developed acute signs of neosporosis when challenged with the highest dose of *N. caninum* NC-Liv tachyzoites. In addition, CBA/Ca mice induced a strong cellular immune response. This mice strain has previously been shown to be suitable for tissue cyst production [18], which renders them an interesting model for investigations on protective effects and potential tools for vaccination. In contrast, the use of BALB/c mice, although extensively employed, is debatable due to their inherent Th2 bias, resulting in increased susceptibility to *N. caninum*. Highly variable results have been obtained using C57BL/6 mice as model. Thus, there is clearly a need for a standardisation of experimental murine infection models for *N. caninum* in order to achieve comparable results between research groups that work with different tools for immunoprevention and chemotherapy. 

## Figures and Tables

**Table 1:** IgG2a or IgG2c and IgG1 titres were measured at the time of death or euthanasia (intercurrent death) or at the end of the experiment at day 34 PI. For calculation, the value “4.5” was chosen when the titre was below the detection limit (<5.0).

Experimental groups				Antibodies at day 34 or time of euthanization (2log)		
		Mouse number	Survival time (days)			
Inbred strain	Challenge dose			IgG2a/c	IgG1	IgG2a/c-IgG1
		1.1	34	13.6	11.2	2.4
		1.2	34	14.1	11.0	3.1
BALB/c	1 × 10^6^	1.3	34	13.0	11.6	1.4
		1.4	34	14.1	14.4	−0.3
		1.5	34	14.7	15.4	−0.7

		2.1	34	14.6	14.6	0.0
		2.2	34	14.8	15.8	−1.0
BALB/c	5 × 10^6^	2.3	34	14.5	14.7	−0.2
		2.4	23	14.4	13.3	1.1
		2.5	23	15.0	14.0	1.0

		3.1	8	—	—	—
		3.2	12	—	—	—
BALB/c	25 × 10^6^	3.3	8	9.9	7.7	2.2
		3.4	12	9.4	8.3	1.1
		3.5	7	—	—	—

		4.1	34	10.8	4.5	6.3
		4.2	34	14.8	6.5	8.3
CBA/Ca	1 × 10^6^	4.3	34	11.0	6.7	4.3
		4.4	34	11.2	4.5	6.7
		4.5	34	13.9	9.8	4.1

		5.1	34	12.2	6.0	6.2
		5.2	34	13.1	5.1	8.0
CBA/Ca	5 × 10^6^	5.3	34	13.3	9.2	4.1
		5.4	34	13.5	9.6	3.9
		5.5	34	13.6	12.2	1.4

		6.1	9	5.0	4.5	0.5
		6.2	9	6.0	6.9	−0.9
CBA/Ca	25 × 10^6^	6.3	8	—	—	—
		6.4	9	5.7	4.5	1.2
		6.5	9	4.5	4.5	0.0

		7.1	34	13.4	9.8	3.6
		7.2	34	14.9	14.6	0.3
C57BL/6	1 × 10^6^	7.3	29	14.1	10.8	3.3
		7.4	29	12.9	7.1	5.8
		7.5	34	14.6	9.2	5.4

		8.1	13	10.1	4.5	5.6
		8.2	7	4.5	4.5	0.0
C57BL6	5 × 10^6^	8.3	34	15.6	13.4	2.2
		8.4	8	10.5	4.5	6.0
		8.5	34	16.5	13.7	2.8

		9.1	8	10.7	6.9	3.8
		9.2	8	7.4	4.5	2.9
C57BL6	25 × 10^6^	9.3	34	15.6	14.5	1.1
		9.4	8	7.7	4.5	3.2
		9.5	8	8.5	4.5	4.0

**Table 2:** Experimental groups, numbers of symptomatic mice, and numbers of parasites in brain and brain lesion scores at the time of death or euthanasia (intercurrent death) or at the end of the experiment at day 34 PI. The detection limit of the PCR is 10 parasites/reaction. Lesions were graded using the following system: (0) no lesions, (1) light positive or small foci, (2) moderately positive, (3) severely positive with large inflammatory process, ∗tachyzoites, and ∗∗cyst.

	Experimental groups (*n* = 5)	Number of symptomatic mice	Time of death (day PI)	Number of parasites in 20 ng brain DNA (real-time PCR)	Brain lesion score (histopathology)
	1 × 10^6^	0	34, 34, 34, 34, 34	0, 0, 0, 361, 300	0, 0, 0, 1, 0
BALB/c	5 × 10^6^	5	34, 34, 34, 34, 23	464, 651, 488, 902, 1183	0, 2, 1, 1, 2
	25 × 10^6^	5	8, 12, 8, 12, 7	587, 763, 260, ND, 154	0, 0, 0, 0, 0

	1 × 10^6^	0	34, 34, 34, 34, 34	0, 0, 0, 0, 156	3∗, 0, 0, 2, 0
CBA/Ca	5 × 10^6^	0	34, 34, 34, 34, 34	0, 0, 0, 0, 0	0, 0, 0, 0, 0
	25 × 10^6^	5	9, 9, 8, 9, 9	466, 433, 349, 606, 593	0, 0, 1, 1, 2

	1 × 10^6^	2	34, 34, 29, 29, 34	0, 177, 525, 0, 763	0, 0, 1, 1, 2
C57BL/6	5 × 10^6^	5	13, 7, 34, 8, 34	312, 0, 638, 0, 879	3∗∗, 0, 2, 0, 3
	25 × 10^6^	5	8, 8, 34, 8, 8	344, 325, 788, 232, 0	0, 0, 2, 0, 0
